# Postoperative sore throat and subglottic injury after McGrath® MAC videolaryngoscopic intubation with versus without a stylet in patients with a high Mallampati score: a randomized controlled trial

**DOI:** 10.1186/s12871-019-0811-x

**Published:** 2019-07-31

**Authors:** Hyun-Kyu Yoon, Hyung-Chul Lee, Hyongmin Oh, Kwanghoon Jun, Hee-Pyoung Park

**Affiliations:** Department of Anesthesiology and Pain Medicine, Seoul National University College of Medicine, Seoul National University Hospital, 101 Daehak-ro, Jongno-gu, Seoul, 03080 South Korea

**Keywords:** McGrath® MAC videolaryngoscopic intubation, Stylet, Postoperative sore throat, Subglottic injury

## Abstract

**Background:**

A tracheal tube stylet can be used to assist successful tracheal intubation, especially during videolaryngoscopic intubation because videolaryngoscopes with a Macintosh-type blade such as McGrath® MAC videolaryngoscope have more acute angle than conventional Macintosh laryngoscope. However, the use of a stylet during tracheal intubation can raise concerns about stylet-induced postoperative airway complications, such as sore throat, subglottic injury, and hoarseness. In this study, we compared the incidence of postoperative airway complications after McGrath® MAC videolaryngoscopic intubation with versus without a stylet in patients with a high Mallampati score.

**Methods:**

In 104 patients with Mallampati score III or IV and who were scheduled for lumbar or thoracic spine surgery, McGrath® MAC videolaryngoscopic intubation was performed either with a stylet (group S, *n* = 52) or without a stylet (group N, n = 52). The primary outcome measure was the incidences of sore throat evaluated at 1 and 24 h postoperatively. Secondary outcome measures were the incidences of subglottic injury and postoperative hoarseness.

**Results:**

The incidence of CL grade III in group S and N was 3.8 and 5.8%, respectively. No patient showed CL grade IV. The incidences of sore throat at 1 (26.9 vs 19.2%, *P* = 0.485) and 24 h (17.3 vs 13.5%, *P* = 0.786, respectively) postoperatively were not significantly different between the group S and N. However, the incidence of subglottic injury was significantly higher in the group S, compared with the group N (65.4 vs 42.3%, *P* = 0.030). The incidence of postoperative hoarseness did not differ significantly between the two groups.

**Conclusions:**

The use of a stylet during McGrath® MAC videolaryngoscopic intubation did not have a significant impact on the incidence of postoperative sore throat in patients with a high Mallampati score. Avoiding the use of a stylet during intubation with McGrath® MAC videolaryngoscope may reduce the incidence of subglottic injury in such patients.

**Trial registration:**

Clinical Research Information Service (identifier: KCT0002427, date of registration: June 12, 2017).

## Background

In clinical practice, a tracheal tube stylet is widely used for difficult airway management. In addition, it can be used to assist successful tracheal intubation, especially during videolaryngoscopic intubation because videolaryngoscopes with a Macintosh-type blade such as McGrath® MAC videolaryngoscope (McGrath® MAC; Aircraft Medical Ltd., Edinburgh, UK) have more acute angle than conventional Macintosh laryngoscope. However, the use of a stylet during tracheal intubation raises concerns regarding stylet-related complications, such as palatal perforation, oropharyngeal injury, subglottic injury, and postoperative pharyngeal pain [1–4].

Although postoperative sore throat usually resolves within a week, it was considered as one of the leading patient complaints after tracheal intubation [5]. In a previous study, tracheal intubation without a stylet during direct laryngoscopy significantly reduced the incidence of postoperative pharyngeal pain, which implied that the application of a stylet itself could affect the development of postoperative sore throat [4]. In cases of videolaryngoscopic intubation, a prior investigation reported that repeated attempts with a styletted endotracheal tube during tracheal intubation with GlideScope® (Verathon Medical, Bothell, WA, USA) significantly increased the incidence of postoperative sore throat in patients with normal airway [6]. Also, one small prospective study conducted by Shimazaki and co-workers showed positive correlation between the use of a stylet during McGrath® MAC videolaryngoscopic intubation and postoperative sore throat in patients without difficult airway [7]. However, the primary outcome was not the incidence of postoperative sore throat in both studies. Furthermore, in the former study, the portion of patients with a high Mallampati score was small, while in the latter study, the Mallampati score was not described. Therefore, it was necessary to investigate the relationship between the use of a stylet during videolaryngoscopic intubation and postoperative sore throat in patients with a high Mallampati score.

In this study, we hypothesized that McGrath® MAC videolaryngoscopic intubation would result in a different postoperative incidence of sore throat, depending on whether a malleable stylet is used or not, in patients with a high Mallampati score. This hypothesis was evaluated by comparing the incidences of postoperative sore throat and subglottic injury in McGrath® MAC videolaryngoscopic intubations with versus without a stylet.

## Methods

### Study population

The institutional review board of Seoul National University Hospital approved this study (1705–071-854, Seoul, Korea), and the study protocol was registered at cris.nih.go.kr (identifier: KCT0002427, June 12, 2017). Written informed consent was obtained from all patients before enrollment. This study was conducted in compliance with Good Clinical Practice Guidelines and adhered to the applicable Consolidated Standards of Reporting Trials (CONSORT) guidelines. Patients between the age of 20 and 80 years with ASA physical status I–III and a modified Mallampati classification III or IV and who were scheduled for elective lumbar or thoracic spine surgery between September 1, 2017 and May 31, 2018 were eligible for this study. Patients who had the following features were excluded: a previous history of radiation therapy or surgery on the airway, coagulopathy, loose teeth, or congenital or acquired upper airway lesions (i.e., tumor, polyp, trauma, abscess, and inflammation). In addition, patients who were deemed to be at increased risk for aspiration during tracheal intubation, such as a gastroesophageal reflux disease were excluded.

### Randomization

Block randomization (a mixture of 13 blocks with six patients per block and eight blocks with four patients per block) was performed to reduce bias and achieve balance in the allocation of participants to two treatment arms using a computer-generated program by an investigator blinded to the study. The allocation order was concealed in opaque envelopes, and it was disclosed by the anesthesia nurse immediately before anesthetic induction. The patients were randomly allocated to the two groups at a 1:1 ratio, and patients, surgeons, and investigators were blinded to the group assignment.

### Study protocol

On the day before surgery, the patient’s airway was evaluated using a modified Mallampati classification and airway evaluation parameters (inter-incisor distance, thyromental distance, thyromental height, and sternomental distance) based on the methods previously described [8, 9]. All patients entered the operating room without any premedication. After basic monitoring devices (three-lead ECG, pulse oximetry, non-invasive blood pressure, and the bispectral index) were connected to the patients, anesthesia was induced with remifentanil and propofol target-controlled infusions (target effect-site concentrations of 4 ng.ml^− 1^ and 4 μg.ml^− 1^, respectively). After the loss of responses to verbal commands, rocuronium (0.6–0.8 mg.kg^− 1^) was administered to facilitate tracheal intubation. When a train-of-four count of 0 was confirmed in the neuromuscular monitoring device (TOF-Watch SX, Bluestar Enterprises, Omaha, NE, USA), tracheal intubation was accomplished by one of two attending anesthesiologists.

All tracheal intubations were performed under indirect vision through the screen display by anesthesiologists who had experiences of ≥30 successful McGrath® MAC videolaryngoscopic intubation, and a pillow with a height of 6–8 cm was used in all patients. In the stylet group (Group S), McGrath® MAC videolaryngoscopic intubation was performed with a malleable aluminum stylet. This stylet was lubricated and bent into a “hockey-stick” curvature and preloaded in the endotracheal tube [10]. The tip of a stylet did not protrude beyond the tip of the endotracheal tube. After positioning the videolaryngoscope at the vallecular fossa, a styletted endotracheal tube was closely introduced to the glottis. Before inserting a styletted endotracheal tube into the glottis, a stylet was slowly removed just in the front of the vocal cord inlet and only the endotracheal tube was advanced into the glottis. In the non-stylet group (group N), McGrath® MAC videolaryngoscopic intubation was performed without a stylet. Despite the optimal videolaryngoscopic view, if there was a significant difficulty in advancing the endotracheal tube into the glottis due to the anteriorly located larynx, the tip of the McGrath® MAC videolaryngoscope was withdrawn slightly from the vallecula fossa to facilitate the advancement of the endotracheal tube. Thereafter, the tongue base was lifted anteriorly. Mallinckrodt wire-reinforced tubes (Medtronic, Dublin, Ireland; internal diameter of 7.5 mm for men and 7.0 mm for women) and size 3 blade of the McGrath® MAC videolaryngoscope were used in both groups.

The intubation time was defined as the interval between insertion of the blade into the oral cavity and withdrawal of the blade from the oral cavity. Visualization of the glottis was assessed based on the Cormack-Lehane (CL) grade under indirect vision when the tip of the McGrath® MAC videolaryngoscope blade was placed at the vallecular fossa to obtain an optimal glottic view [11]. The success of tracheal intubation was confirmed by end-tidal carbon dioxide monitoring with capnography. Heart rate and mean arterial pressure were recorded just before and 1 min after tracheal intubation.

In both groups, an intubation time of > 2 min was regarded as a failed intubation attempt. If the first attempt failed, further attempts were made by the same anesthesiologist after the patient had undergone 1 min of mask ventilation with oxygen. If oxygen saturation was < 90% during tracheal intubation, the procedure was stopped and mask ventilation was resumed until recovery of oxygen saturation. A maximum of three attempts were allowed. If the third attempt also resulted in failure, a lighted stylet was used for successful tracheal intubation. The cuff pressure of the endotracheal tube was measured using a Posey 8199 Cufflator™ (Posey Company, Arcadia, CA, USA) just after tracheal intubation and a positional change, and it was maintained within 25 cm H_2_O during the rest of surgery. The optimal depth of placement of the endotracheal tube was determined by palpating suprasternal notch.

At the end of surgery, the fiberoptic bronchoscope was introduced through the endotracheal tube before emergence. After pulling out the endotracheal tube to the proximal end of insertion cord of the bronchoscope, fiberoptic bronchoscopic examination was performed on the trachea and larynx to assess the grade of the subglottic injury. Thereafter, manual ventilation using facial mask was performed until full recovery from anesthesia. The degree of subglottic injury was expressed as four grades (none: no subglottic injury, mild: mucosal hyperemia and edema or slight submucosal hematoma, moderate: moderate submucosal hematoma, or severe: mucosal laceration and/or mucosal bleeding) [3], and it was evaluated by one of two anesthesiologists who were not involved in the intubation procedure and blinded to the use of the stylet. Blood in the oral cavity and blood staining on the endotracheal tube were also recorded. The presence or absence of sore throat and hoarseness was evaluated at 1 and 24 h postoperatively by an investigator blinded to this study. Sore throat was assessed with numeric rating scale from 0 to 10 (0: no sore throat, 10: the worst imaginable pain).

### Study outcomes

The primary outcome measure of this study was the incidence of postoperative sore throat. Secondary outcome measures were the incidences of postoperative hoarseness, blood in oral cavity, and blood staining on the endotracheal tube, the intubation time, the success rate of tracheal intubation, the degree of subglottic injury evaluated by the fiberoptic bronchoscope, and hemodynamic variables (mean arterial pressure and heart rate) before and 1 min after tracheal intubation.

### Statistical analysis

The incidence of postoperative sore throat was compared using the chi-squared test. Other categorical variables, including the incidences of postoperative hoarseness, blood in oral cavity, blood staining on the endotracheal tube, the success rate of tracheal intubation, and the degree of subglottic injury were compared using the chi-squared test or Fisher’s exact test. The intubation time was compared using the Student’s *t*-test. Hemodynamic variables were analyzed using repeated measures analysis of variance to determine a group-by-time interaction effect and the values at each time point were compared using the Student’s *t*-test with Bonferroni correction. All statistical analyses were performed using SPSS software (version 25.0; IBM Corp., Armonk, NY, USA). A *P* value < 0.05 was considered to indicate a statistical significance.

### Sample size calculation

Previous studies reported that the incidence of postoperative sore throat after McGrath® MAC videolaryngoscopic intubation with a stylet was 9–45.4% with an average of about 25% [12–15]. To test the ability of McGrath® MAC videolaryngoscopic intubation without a stylet to reduce this incidence to 5%, at least 49 patients were enrolled in each group, based on an alpha of 0.05 (two-tailed) and a beta of 0.2. Taking into consideration of a possible dropout rate of 5%, a total of 110 patients were enrolled in this study.

## Results

**Fig. 1:**
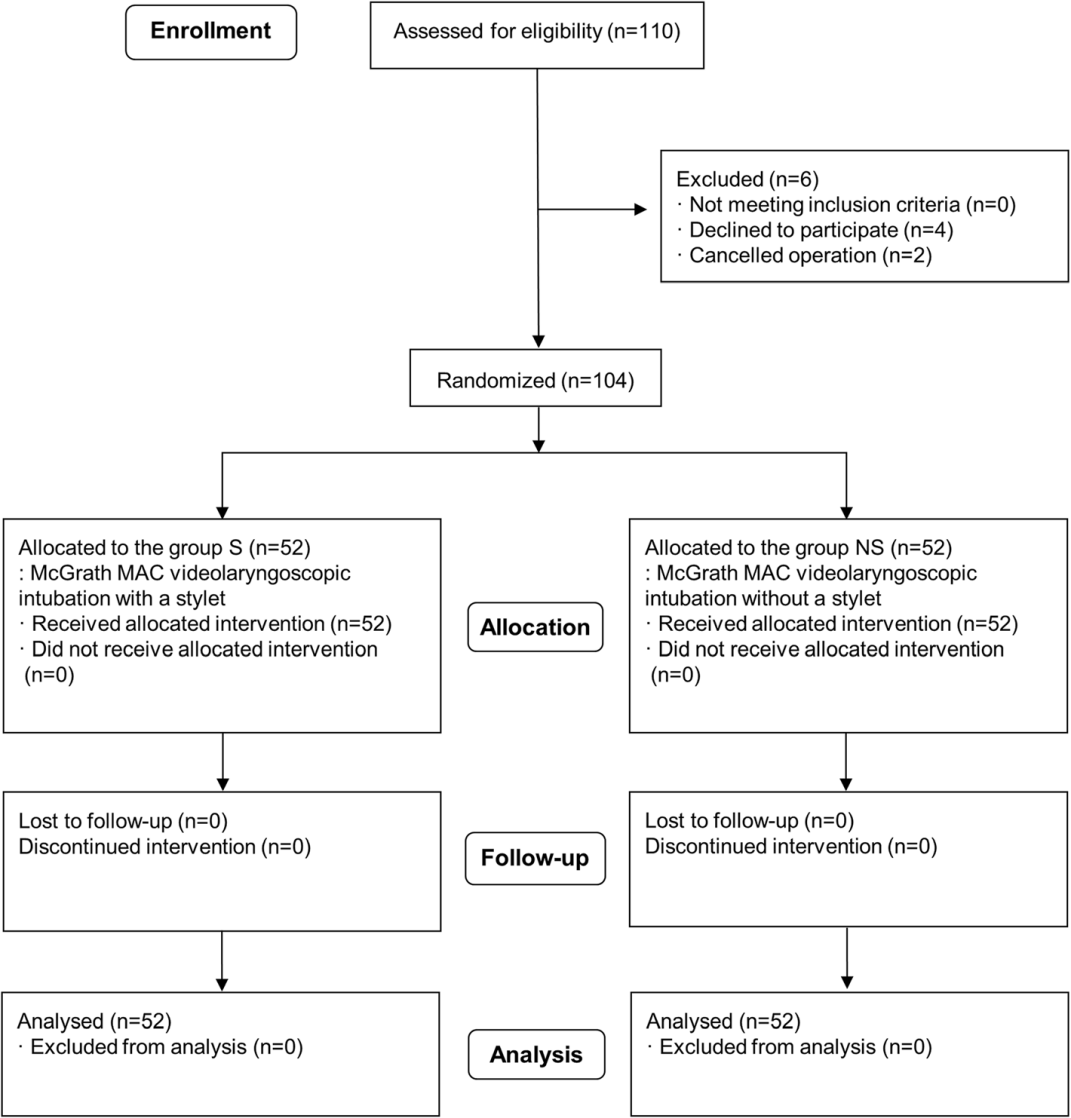
CONSORT flow diagram of the study

Of the 110 patients eligible for the study, six patients were excluded due to the cancelled operation and refusal of participation (Fig. 1). There were no significant differences in baseline characteristics and airway-related variables between the group S and N (Table 1).

**Table 1:** Comparisons of demographics and airway-related variables between the two groups

Parameters	Group S (*n* = 52)	Group N (*n* = 52)	Mean Difference (95% CI)
Age (years)	58.1 ± 14.8	60.8 ± 15.0	2.7 (− 3.1 to 8.5)
Male sex	25 (48.1%)	25 (48.1%)	0.0% (−18.5 to 18.5)
BMI (kg.m^−2^)	25.5 ± 4.2	25.3 ± 3.4	0.2 (− 1.7 to 1.3)
ASA PS classification			
I	20 (38.5%)	19 (36.5%)	2.0% (−16.1 to 19.9)
II	27 (51.9%)	26 (50.0%)	1.9% (−16.7 to 20.3)
III	5 (9.6%)	7 (13.5%)	3.9% (−9.0 to 16.9)
Comorbidities			
Hypertension	19 (36.5%)	19 (36.5%)	0.0% (−18.0 to 18.0)
Diabetes mellitus	3 (5.8%)	5 (9.6%)	3.8% (−7.5 to 15.4)
Cardiac disease	3 (5.8%)	3 (5.8%)	0.0% (−10.6 to 10.6)
Respiratory disease	3 (5.8%)	5 (9.6%)	3.8% (−7.5 to 15.4)
Neurologic disease	6 (11.5%)	6 (11.5%)	0.0% (−13.0 to 13.0)
Renal disease	2 (3.8%)	0 (0.0%)	3.8% (−3.6 to 12.9)
Hepatic disease	3 (5.8%)	3 (5.8%)	0.0% (−10.6 to 10.6)
Thyroid disease	0 (0.0%)	1 (1.9%)	1.9% (− 5.2 to 10.1)
Malignancy	4 (7.7%)	3 (5.8%)	1.9% (−9.0 to 13.1)
Airway evaluations			
Mallampati score			
III	48 (92.3%)	47 (90.4%)	1.9% (−9.9 to 13.8)
IV	4 (7.7%)	5 (9.6%)	1.9% (−9.9 to 13.8)
Inter-incisor distance (mm)	42.7 ± 9.8	40.6 ± 8.0	2.1 (−5.6 to 1.4)
Thyromental distance (mm)	73.3 ± 16.2	70.6 ± 14.5	2.7 (−8.7 to 3.3)
Thyromental height (mm)	52.2 ± 13.1	49.8 ± 11.1	2.4 (−7.1 to 2.3)
Sternomental distance (mm)	138.0 ± 26.5	137.5 ± 26.8	0.5 (−10.9 to 9.9)
Retrognathia	0 (0.0%)	2 (3.8%)	3.8% (−3.6 to 12.9)
Anesthesia time (min)	221.1 ± 149.8	212.8 ± 114.6	8.3 (−60.2 to 43.6)
Operation time (min)	165.4 ± 143.2	159.5 ± 109.1	5.9 (−55.4 to 43.6)

Data are presented as mean ± SD or number of patients (%)

*CI* confidence interval, *ASA PS* American Society of Anesthesiologists physical status classification. In the group S, McGrath® MAC videolaryngoscopic intubation was performed with a stylet. In the group N, McGrath® MAC videolaryngoscopic intubation was performed without a stylet

The incidence of sore throat at 1 (26.9 vs 19.2%, *P* = 0.485) and 24 h (17.3 vs 13.5%, *P* = 0.786) postoperatively did not significantly differ between the two groups (Table 2). In addition, there were no significant differences in the incidence of hoarseness at 1 (17.3 vs 7.7%, *P* = 0.235) and 24 h (3.8 vs 7.7%, *P* = 0.678) postoperatively. However, with respect to subglottic injury, the overall incidence of subglottic injury and the incidence of mild subglottic injury were significantly higher in the group S, compared with the group N (65.4 vs 42.3%, *P* = 0.030; 53.8 vs 32.7%, *P* = 0.048, respectively).

**Table 2:** Comparisons of postoperative airway complications between the two groups

Complications	Group S (*n* = 52)	Group N (*n* = 52)^*^	Mean Difference (95% CI)	*P* value
Sore throat				
Postoperative 1 h	14 (26.9%)	10 (19.2%)	7.7% (−8.5 to 23.5%)	0.485
Postoperative 24 h	9 (17.3%)	7 (13.5%)	3.8% (−10.4 to 18.0%)	0.786
Sore throat ^*a*^				
Postoperative 1 h	4.5 (3.0–7.0)	4.5 (3.0–5.3)	NA	0.807
Postoperative 24 h	5.0 (3.0–6.0)	5.0 (3.0–6.0)	NA	0.905
Hoarseness				
Postoperative 1 h	9 (17.3%)	4 (7.7%)	9.6% (−3.5 to 22.9%)	0.235
Postoperative 24 h	2 (3.8%)	4 (7.7%)	3.9% (−6.3 to 14.7%)	0.678
Blood in oral cavity	4 (7.7%)	2 (3.8%)	3.9% (−6.3 to 14.7%)	0.678
Blood staining on the endotracheal tube	4 (7.7%)	2 (3.8%)	3.9% (−6.3 to 14.7%)	0.678
Degree of subglottic injury				
Overall	34 (65.4%)	22 (42.3%)	23.1% (4.0 to 40.0%)	0.030
Mild	28 (53.8%)	17 (32.7%)	21.1% (2.1 to 38.1%)	0.048
Moderate	4 (7.7%)	5 (9.6%)	1.9% (−9.9 to 13.8%)	1.000
Severe	2 (3.8%)	0 (0.0%)	3.8% (−3.6 to 12.9)	0.495

Data are presented as number of patients (%) or median (interquartile range)

*CI* confidence interval, *NA* not applicable

* In the group N, one patient’s trachea was not intubated even after three intubation attempts

^*a*^ Sore throat was evaluated with numeric rating scale from 0 to 10 (0: no pain, 10: the worst imaginable pain) and compared using the Mann-Whitney *U* test

In the group S, McGrath® MAC videolaryngoscopic intubation was performed with a stylet. In the group N, McGrath® MAC videolaryngoscopic intubation was performed without a stylet

Comparisons of the intubation-related variables between the two groups are presented in Table 3. The incidence of Cormack-Lehane (CL) grade III in group S and N was 3.8 and 5.8%, respectively. No patient showed CL grade IV. The initial success rate of tracheal intubation and intubation time were comparable between the two groups. Only one patient’s trachea in the group N was not intubated even after three intubation attempts, because the inlet of the vocal cord was not visible due to the anteriorly located larynx. The trachea of this patient was intubated successfully by using McGrath® MAC videolaryngoscopy-assisted a lighted stylet with the classic “hockey-stick” shape. During emergence, there were no airway adverse events such as laryngospasm and oxygen desaturation (< 90% of SpO_2_).

**Table 3:** Comparisons of intubation-related variables between the two groups

Parameters	Group S (*n* = 52)	Group N (*n* = 52)	Mean Difference (95% CI)	*P* value
Successful tracheal intubation				
At first attempt	52 (100.0%)	51 (98.1%)	1.9% (− 5.2 to 10.1)	1.000
At second attempt	0 (0.0%)	0 (0.0%)	NA	NA
At third attempt	0 (0.0%)	0 (0.0%)	NA	NA
CL grade at optimal view				
I	40 (76.9%)	37 (71.2%)	5.7% (−11.1 to 22.1%)	0.655
II	10 (19.2%)	12 (23.1%)	3.9% (−11.8 to 19.4%)	0.810
III	2 (3.8%)	3 (5.8%)	2.0% (−7.9 to 12.3%)	1.000
IV	0 (0.0%)	0 (0.0%)	NA	NA
Intubation time (s)	21.8 ± 13.0	22.9 ± 14.3	1.1 (−4.2 to 6.4)	0.680
Mean arterial pressure (mmHg)				
Before intubation	77.0 ± 18.0	74.5 ± 16.5	2.5 (−9.2 to 4.2)	0.469
1 min after intubation	97.5 ± 23.1	91.5 ± 21.6	6.0 (−14.7 to 2.7)	0.175
Heart rate (beats/min)				
Before intubation	65.8 ± 12.2	65.1 ± 12.3	0.7 (−5.5 to 4.1)	0.782
1 min after intubation	80.7 ± 15.8	80.1 ± 16.6	0.6 (−6.9 to 5.7)	0.853

Data are shown as number (%) or mean ± SD

*CI* confidence interval, *NA* not applicable, *CL grade* Cormack-Lehane grade

In the group S, McGrath® MAC videolaryngoscopic intubation was performed with a stylet. In the group N, McGrath® MAC videolaryngoscopic intubation was performed without a stylet

## Discussion

In this study, McGrath® MAC videolaryngoscopic intubation without a stylet did not significantly decrease the incidences of postoperative sore throat and hoarseness. However, the overall incidence of subglottic injury was significantly decreased when a stylet was not used during McGrath® MAC videolaryngoscopic intubation.

As postoperative sore throat is usually self-limiting and improves within a week, it is generally considered as a minor complication. The incidence of postoperative sore throat after McGrath® MAC videolaryngoscopic intubation was reported to be 5–45.4% [7, 12–18], and its causes were multifactorial. In this study, when McGrath® MAC videolaryngoscope with size 3 MAC blade was used for tracheal intubation, no significant difference in the incidence of postoperative sore throat was observed between patients with the use of a malleable “hockey-stick” curved stylet and those without. This finding might be explained by the following reasons. First, postoperative sore throat may correlate with the severity of subglottic injury. That is, mild subglottic injury can have little impact on the development of postoperative sore throat, while moderate or severe subglottic injury can have a significant impact. In the stylet group, although the incidence of mild subglottic injury was as high as 54%, the incidence of moderate and severe subglottic injury was low (12%). This could be attributed to the cautious manipulation of the lubricated stylet to prevent unintended mucosal injury to upper airway structures and to its gentle removal to prevent further subglottic injury caused by excessive extraction force. Second, other factors affecting the development of postoperative sore throat, such as the size and cuff pressure of the endotracheal tube, were well-controlled in this study. A relatively small wire-reinforced tube (internal diameter of 7.5 mm for men and 7.0 mm for women) was used, and the cuff pressure was maintained at 25 cm H_2_O during the surgery, which may result in no significant difference in the incidence of postoperative sore throat between the two groups. Furthermore, a stylet in the group S was cautiously removed in the front of the vocal cord inlet to prevent stylet-induced direct subglottic mucosal injury, which is a relevant cause of postoperative sore throat.

Among intubation-related airway complications, subglottic injury can occur by a tracheal tube itself. The stiffened tip of an endotracheal tube can cause subglottic injury to the anterior tracheal wall after the endotracheal tube passes through the glottis. In addition, when a stylet is removed, the endotracheal tube starts to curve anteriorly, which can increase the risk of subglottic injury by hitting the anterior tracheal wall [3, 19, 20]. In this study, McGrath® MAC videolaryngoscopic intubation with a stylet resulted in a higher incidence of subglottic injury, compared with those without. However, the differences in the initial success rate of tracheal intubation, the intubation time, and hemodynamic changes were not statistically significant between the two groups.

In the non-stylet group, the initial success rate of tracheal intubation was 98.1%. When a non-styletted endotracheal tube was not directed to the glottis due to the anteriorly located larynx, withdrawal of the videolaryngoscope blade from the vallecular fossa reduced the introduction angle of the endotracheal tube to the glottis such that its curvature more resembled that of a direct laryngoscope. This change facilitated the insertion of a malleable wire-reinforced tube. Similarly, a previous literature review suggested that when the glottis was visualized well but insertion and advancement of the endotracheal tube failed during videolaryngoscopic intubation, withdrawal of the GlideScope® blade might be helpful in increasing the success rate of tracheal intubation without a stylet by decreasing the introduction angle of the endotracheal tube in some patients [21]. In the present study, one patient in the non-stylet group showed intubation failure even after three intubation attempts because the larynx of the patient was located too anteriorly. In this patient, the introduction angle of the endotracheal tube was still acute, although the angle was slightly reduced by withdrawing the laryngoscope blade. In this situation, considering the use of a stylet or a lighted stylet with a “hockey-stick” shape may be reasonable for successful tracheal intubation.

McGrath® MAC videolaryngoscope was chosen for this study due to several advantages over other videolaryngoscopes. First, the blade curvature of McGrath® MAC videolaryngoscope is similar to that of the Macintosh laryngoscope, which could offer familiarity to practitioners with the experience of Macintosh laryngoscopic intubation. Second, in videolaryngoscopic intubation with a Macintosh-type blade, the use of a stylet is not necessary for successful tracheal intubation in patients with normal airway [22]. Lastly, McGrath® MAC videolaryngoscope showed higher success rates of tracheal intubation and lower rates of tissue trauma to airway structures than other videolaryngoscopes in patients with simulated difficult airway [14].

This study had several limitations. First, the attending anesthesiologist was not blinded during McGrath® MAC videolaryngoscopic intubation, which may have resulted in biases, although the data on postoperative airway complications were collected by an anesthesiologist who was blinded to the group assignment. Second, because postoperative sore throat may be somewhat subjective, biases in the recorded incidence may have occurred. Third, only McGrath® MAC videolaryngoscope was selected for tracheal intubation in this study. McGrath® X-blade videolaryngoscope, which has a hyper-angulated blade, can be more appropriate for difficult airway management because it can provide a better glottic view in such situation. Also, the malleable stylet used in this study may be not helpful with McGrath® MAC videolaryngoscope. The movement given to the proximal end of the endotracheal tube to get the glottic image on the monitor is often not transmitted to its distal tip due to deformation of the stylet. Therefore, it is difficult to apply our intubation methods to patients with difficult airway. Fourth, although only patients with a modified Mallampati score III or IV were enrolled in this study, most patients did not have a difficult airway. Actually, the incidence of CL grade III in group S and N was 3.8 and 5.8%, respectively. No patient showed CL grade IV. A recent meta-analysis revealed that Mallampati classification was not appropriate as a single test of a difficult laryngoscopy [23]. Therefore, there is a limitation in extrapolating our results to patients with difficult airway. Finally, the Mallinckrodt wire-reinforced tube was used in all tracheal intubations because all surgeries in this study were performed in the prone position. However, numerous endotracheal tubes differing in their geometry (body curvature, flexibility, and shape of the endotracheal tube tip) have been used in clinical practice. Two previous reports showed that the type of an endotracheal tube influenced the occurrence of subglottic injury during tracheal intubation using GlideScope® and C-MAC® videolaryngoscopes, due to differences in the degree of deformability of the endotracheal tube tip [3, 20]. Therefore, caution is needed in interpreting our results.

## Conclusions

In conclusion, this study showed that the use of a stylet during McGrath® MAC videolaryngoscopic intubation did not significantly affect the development of postoperative sore throat in patients with Mallampati score III or IV. Omitting the use of a stylet during McGrath® MAC videolaryngoscopic intubation may reduce the incidence of subglottic injury in such patients.

## Data Availability

The datasets created during and/or analyzed during the current study are available from the corresponding author on reasonable request.

## Abbreviations

CL grade: Cormack-Lehane grade
CONSORT: Consolidated Standards of Reporting Trials
